# Biological response to hormonal manipulation in oestrogen receptor positive ductal carcinoma *in situ* of the breast

**DOI:** 10.1038/sj.bjc.6601013

**Published:** 2003-07-15

**Authors:** G P Boland, A Mckeown, K C Chan, R Prasad, W F Knox, N J Bundred

**Affiliations:** 1 Department of Surgery, University Hospital of South Manchester, Manchester M20 8LR, UK; 2 Department of Pathology, University Hospital of South Manchester, Manchester M20 8LR, UK

**Keywords:** DCIS, HRT, response, tamoxifen, recurrence, breast

## Abstract

Adjuvant antioestrogen therapy with tamoxifen is recommended for all women following breast-conserving surgery for ductal carcinoma *in situ* (DCIS) to reduce local recurrence, despite 50% of lesions being oestrogen receptor (OR) negative. We have investigated the response to hormone manipulation in DCIS by studying changes in epithelial proliferation and progesterone receptor (PR) expression as surrogate molecular markers of treatment effects in DCIS of known OR status. Women were identified who had undergone diagnostic core biopsy followed by surgery for DCIS 14–41 days later. Ki67 (a measure of epithelial cell proliferation) and PR expression were determined by immunohistochemistry on paired paraffin sections of the core biopsy and operative specimens for each patient, with OR and HER-2 measured on the operative specimen. Women were divided into three groups according to whether they had changed hormone therapy (stopped hormone replacement therapy (HRT), group 1), continued taking HRT (group 2) or were not taking HRT (group 3) between core biopsy and surgery. In OR-positive (but not in OR-negative) DCIS after oestrogen withdrawal (group 1), a fall in the mean cell proliferation (*P*<0.01) was observed. A fall in PR expression between core biopsy and surgery was also seen in this group (*P*=0.02). No change in either mean cell proliferation or PR expression was seen in the other two groups in OR-positive or -negative DCIS. The fall in proliferation and PR expression occurred regardless of HER-2 status. In conclusion, a biological response to hormone manipulation is only seen in OR-positive DCIS tumours. Any clinical value of antioestrogen therapy is likely to be restricted to this group.

Ductal carcinoma *in situ* (DCIS) accounts for 15–20% of new breast cancers and up to 40% of mammographic detected cancers (Ernster *et al*, 2000). Most patients with DCIS are now offered breast-conserving surgery (BCS) (Bordeleau *et al*, 2001) in marked contrast to the practice two decades ago when mastectomy was the standard treatment. Breast-conserving surgery, even with the addition of adjuvant radiotherapy, is associated with local recurrence (LR) rates of up to 10% at 4 years (Fisher ER *et al*, 1999; George *et al*, 2000; Julien *et al*, 2000) compared to 1–2% after mastectomy, with about half of all recurrences being invasive cancers (Solin *et al*, 2001).

Breast epithelial proliferation is increased by the use of the oral contraceptive pill (Williams *et al*, 1991) and hormone replacement therapy (HRT) use exceeding 5 years duration (Hofseth *et al*, 1999), although the role of HRT as a risk factor for the development of DCIS is unclear.

Two studies (Schairer *et al*, 1994; Longnecker *et al*, 1996) have reported a 1.4 increased relative risk (RR) of DCIS development with use of oestrogen-only HRT preparations and 1.7–2.3 with combined oestrogen and progesterone therapy. However, other studies have not shown any increased RR of DCIS, regardless of the type of HRT preparation used or duration of therapy (Stanford *et al*, 1995; Henrich *et al*, 1998; Gapstur *et al*, 1999). There are no data on the risk of HRT use following treatment for DCIS.

The cellular pathogenesis of DCIS is unclear, but the increased cell proliferation of oestrogen receptor (OR) expressing normal breast epithelial cells near menopause (Shoker *et al*, 1999) may induce low-grade OR-positive DCIS development. No cell precursor of OR-negative DCIS has been identified, but potentially it arises from OR-negative luminal epithelial cells.

In the normal breast, epithelial cell coexpression of OR and Ki67 nuclear antigen (a reliable marker of proliferation in breast cancer (Gonzalez-Vela *et al*, 2001)) is rare, that is, the OR expressing normal breast cell population (10%) are nonproliferating. However, in intraepithelial neoplasia, coexpression is significantly increased, and correlates positively with the risk of invasive cancer development (Shoker *et al*, 1999). The proliferation rate in OR-negative DCIS has been shown to be greater than in OR-positive DCIS, and is thought to be due to a transition to autonomous growth, utilising nonhormonal growth pathways (Schmitt, 1995).

Antioestrogen therapy with tamoxifen (NSABP P-1 trial, Fisher *et al*, 1998) and raloxifene (MORE trial, Cummings *et al*, 1999) has been shown to reduce the development of OR-positive, but not OR-negative, invasive breast cancer (IBC). Tamoxifen is a selective OR modulator that blocks the binding of oestrogen to its nuclear receptor, is cytostatic in action (O'Regan and Jordan, 2002) and has been shown to reduce cell proliferation in IBC (Clarke *et al*, 1993) and in normal breast epithelium (Bernardes *et al*, 1999) as determined, using Ki67 nuclear antigen immunohistochemistry. However, tamoxifen induces progesterone receptor (PR) expression for up to 6 weeks after commencing therapy(Clarke *et al*, 1993; Chang *et al*, 2000; Robertson *et al*, 2001).

Two large randomised clinical trials of adjuvant tamoxifen after BCS for DCIS have been completed (Fisher B *et al*, 1999; George *et al*, 2000). In the NSABP B-24 trial (Fisher B *et al*, 1999), women undergoing tamoxifen therapy for DCIS after radiotherapy (30% of women were <50 years of age) had a significant reduction in the number of cancer events compared to placebo in women undergoing surgery and HRT (8.2 *vs* 13.4%) at 5 years, but this was largely due to the 40% reduction in recurrence in women under 50 years of age on tamoxifen, whereas only a 20% reduction was seen in women >50 years of age randomised to tamoxifen. Recent data from a retrospective pathological review of 628 DCIS tumours out of the original 1804 in this trial showed 77% OR positivity; a clear effect of tamoxifen therapy compared to placebo was seen in these tumours, with a reduction in both ipsilateral and contralateral local recurrence (Allred *et al*, 2002). In contrast, no significant benefit of tamoxifen therapy was seen for OR-negative tumours.

The UK DCIS trial (George *et al*, 2000), in which 97% of patients were ⩾50 years of age, found a nonsignificant 20% reduction in risk of DCIS recurrence from the use of tamoxifen as adjuvant therapy in this older population. A pathological review of this trial data is not yet available.

We have shown previously that approximately 50% of high-grade comedo DCIS is OR negative (Holland *et al*, 1997) and in a human DCIS xenograft animal model hormone independent (Gandhi *et al*, 2000). These observations together with the data on the chemoprevention of OR-positive (but not OR-negative) IBC with antioestrogen therapy (Fisher *et al*, 1998; Cummings *et al*, 1999), suggest that there will be no clinical benefit of antioestrogen therapy after treatment for OR-negative DCIS.

Studies of patients with IBC have shown that early changes (<3 weeks) in cell proliferative indices and PR expression occur after antioestrogen therapy (Clarke *et al*, 1993; Chang *et al*, 2000; Dowsett *et al*, 2001a; Robertson *et al*, 2001; Harper-Wynne *et al*, 2002) in OR-positive (but not OR-negative) tumours and these changes are accepted as surrogate markers of clinical tumour response, with tamoxifen treatment increasing (Chang *et al*, 2000; Robertson *et al*, 2001) and aromatase inhibitor therapy decreasing PR expression (Miller *et al*, 2001).

No equivalent studies of hormonal manipulation of DCIS have been reported. We have utilised the widespread use of core biopsy to diagnose DCIS, and compared proliferation rates in the paired core biopsy and operative surgical specimen in women stopping HRT with a control group who either continued HRT or were not taking HRT.

The aim of this study was to investigate the effect of hormone manipulation on epithelial cell proliferation and PR expression in DCIS in relation to OR status of the DCIS tumours, to evaluate whether any likely benefit of giving adjuvant tamoxifen therapy or discontinuing HRT therapy relates to the OR status of the DCIS tumour.

## MATERIALS AND METHODS

Between 1998 and 2000, 108 women had a preoperative breast core biopsy diagnosis of DCIS (using a 14-gauge needle), followed by definitive surgery between 14 and 41 days later (median 17 days) at the University Hospital of South Manchester or the Christie Hospital, UK. Of these, 52 women were using HRT at the time of diagnosis and 56 women were not using HRT during the same time period. These patients form the retrospective study population and were divided into three groups according to HRT use: Group 1 (*n*=27) contained patients who stopped using HRT at core diagnosis; Group 2 consisted of women who continued to take HRT between the core biopsy and surgery (*n*=25); and Group 3 were not using HRT (or any other hormonal therapy) between diagnosis and surgery (*n*=56), and were designated as a control group.

The HRT preparations taken by patients in Groups 1 and 2 were administered orally in 72% and transdermally (patches) in 28%, and contained oestrogen only in 41% and combined oestrogen and progesterone in 59%.

### Immunohistochemical assay of Ki67, OR, PR and HER-2

Paired paraffin wax sections (3–5 *μ*m thick) of tissue from each patient were mounted on 3-aminopropyltriethoxysilane (APES, Sigma, Dorset, UK) coated slides, dewaxed in xylene and rehydrated prior to immunohistochemical staining for Ki67 nuclear antigen labelling (a measure of cellular proliferation), OR, PR and HER-2. Ki67 was detected using the MIB-1 monoclonal antibody. Well-established immunohistochemistry protocols developed at the clinical research laboratory, Paterson Institute for Cancer Research, Manchester (a UK reference laboratory for HER-2 immunohistochemical staining) were followed for each marker, all of which have previously been validated by us and found to be reproducible in DCIS (Holland *et al*, 1997; Gandhi *et al*, 2000; Dowsett *et al*, 2001a; Chan *et al*, 2002). For all markers, antigen retrieval was achieved by the pressure cooking method for 4 min in citrate buffer (pH=6.0). After cooling (20 min), endogenous peroxide activity was blocked by incubating the slides in phosphate-buffered saline (PBS) with 0.3% H_2_O_2_ for 20 min. A positive and negative control slide was included in each immunohistochemical assay. For HER-2, this included sections of known HER-2-positive invasive breast, and sections of DCIS for OR, PR and Ki67 were used as determined in previous studies of DCIS in our department.

### Ki67 MIB-1 labelling

Nonspecific binding was blocked by using 10% normal goat serum and 0.5% casein in PBS with for 30 min. Sections were then incubated with the mouse monoclonal antibody MIB-1 (Dako Ltd, Cambridge, UK M7240) at a concentration of 1 : 50 for 1 h, then rinsed in PBS for 10 min, followed by incubation with a biotinylated goat anti-mouse secondary antibody (Dako, E432) diluted 1 : 200 for 40 min.

### OR labelling

Nonspecific binding was blocked by using 10% normal goat serum and 0.5% casein in PBS with for 30 min. Sections were incubated with mouse anti-human OR (Dako Ltd, UK M7047), at a concentration of 1 : 33 for 1 h, followed by incubation with a biotinylated secondary goat anti-mouse antibody (Dako, E432), at a concentration of 1 : 200 for 40 min.

### PR labelling

Nonspecific binding was blocked by using 10% normal rabbit serum and 0.5%. casein in PBS with for 30 min. Sections were incubated with a mouse anti-human PR (Dako Ltd, UK M3569), at a concentration of 1 : 50 for 1 h, followed by incubation with a biotinylated secondary rabbit anti-mouse antibody (Dako, E413), at a concentration of 1 : 200 for 40 min.

### HER-2 labelling

Nonspecific binding was blocked by using 10% normal rabbit serum and 0.5%. casein in PBS with for 30 min. Sections were incubated with a mouse anti-human HER-2 (Dako Ltd, UK A485), at a concentration of 1 : 40 for 1 h, followed by incubation with a biotinylated secondary rabbit anti-mouse antibody (Dako, E413), at a concentration of 1 : 200 for 40 min.

### Antigen visualisation

For each antigen, following incubation with the secondary antibody, the tissue was then rinsed in PBS followed by incubation with a standard streptavidin–biotin complex (ABC, Vector labs, Peterborough, UK, PK-6100) for 30 min. Staining was then visualised using diaminobenzidene chromogen (DAB, Dako Ltd, Cambridge, UK) in 0.1% H_2_O_2_ PBS solution and sections counterstained with Gill's haematoxylin.

### Immunohistochemical scoring

Immunostaining was nuclear for Ki67, OR and PR and predominantly cell membranous for HER-2 with a cytoplasmic component. For each section, a minimum of 1000 cells were scored across randomly selected areas of DCIS at a magnification of × 400 using a grid graticule and cell counter. Ki67, OR and PR scores were calculated as the percentage of positively DAB-stained nuclei (i.e. positive cells/total number of cells × 100%). Oestrogen receptor and PR positivities were defined as ⩾5% stained nuclei; consistent with that used for IBCs. HER-2 staining was scored 0 (absent) to 3 (maximum cytomembranous staining seen, comparable to invasive cancer control), with a score ⩾2 considered HER-2 positive.

### Statistical analysis

Statistical analysis was performed using SPSS software (SPSS, Chicago, IL, USA). Changes in proliferative indices and receptor expression levels between core biopsy specimens and operative specimen for each subgroup were compared using paired sample *t*-tests. The magnitude of changes in different parameters across different patient groups was compared using the Kruskal–Wallis test. A significance level of 5% was used throughout.

## RESULTS

The median age of the patients in the three groups (Group 1: women stopped HRT; Group 2: patients continued HRT and Group 3: were not using HRT between core biopsy and surgery) was similar: 56 (range 46–77), 56 (range 42–63) and 57 (range 34–83) years, respectively (*P*=0. 70, Kruskal–Wallis test). High nuclear grade DCIS (grade 3) was present in 63, 48 and 66% of tumours in the three groups, respectively (*P*=0.57, Kruskal–Wallis). Despite the lower frequency of high-grade lesions in the HRT continued group (*n*=25), the proportion of OR-positive tumours across the three groups (67, 76 and 57%, respectively) did not differ (*P*=0.27, *χ*^2^ test).

### Effects of hormone manipulation on cell proliferation

In no OR-negative DCIS tumours, irrespective of group, was a change in cell proliferation seen between core biopsied and operative specimen. In particular, in the hormonally manipulated group (HRT stopped), the OR-negative DCIS tumours showed no change in mean cell proliferation between core biopsy and operation (Table 1

**Table 1:** Changes in cell proliferation (Ki67) in DCIS between core biopsy and surgery by OR status

**OR status**	**Patient group**	**% High grade**	**Mean core biopsy Ki67 LI (s.e.)**	**Mean operation Ki67 LI (s.e.)**	**95% CI of the mean difference**	***P*-value^a^**	**Median Ki67 change core biopsy to operation (IQR)**	***P*-value^b^**
Negative (*n*=39)	Stopped HRT (*n*=9)	78	16.94 (3.01)	16.07 (2.13)	−2.09 to 3.83	0.52	−0.45 (−1.76 to +1.84)	
	Continued HRT (*n*=6)	33	10.84 (4.57)	12.72 (4.62)	−6.19 to 2.45	0.32	+0.42 (−0.25 to +3.44)	0.77
	Not using HRT (*n*=24)	67	12.52 (1.62)	11.99 (1.55)	−1.59 to 2.66	0.61	+0.19 (−4.15 to +3.09)	
								
Positive (*n*=69)	Stopped HRT (*n*=18)	56	9.95 (1.89)	5.06 (1.39)	2.07 to 7.71	<0.01	−3.02 (−7.68 to −0.84)	
	Continued HRT (*n*=19)	53	10.27 (1.79)	9.79 (1.72)	−2.64 to 3.60	0.75	−0.60 (−2.94 to +4.47)	0.02
	Not using HRT (*n*=32)	65	10.81 (1.34)	10.31 (1.76)	−2.60 to 3.61	0.74	−0.52 (−2.4 to +0.34)	

a Paired samples *t*-test for change in Ki67 between core biopsy and surgery for each subgroup.

b Kruskal–Wallis test for the magnitude of change in Ki67 across groups. DCIS=ductal carcinoma *in situ*; OR=oestrogen receptor; LI=labelling index (% of nuclei labelled); CI=confidence interval; s.e.=standard error of mean; IQR=interquartile range.

Changes in DCIS cell proliferation (Ki67 labelling index) between core biopsy and surgery (14–41 days later), with women placed in three groups: stopped HRT, continued HRT and not using HRT. For each group, the DCIS tumours are divided into OR positive and OR negative. The mean Ki67 LI score (%) is given for each subgroup at core biopsy and operation along with the standard error of the mean for each, and differences in the means for the paired samples are compared using a *t*-test. Oestrogen receptor-positive DCIS in the HRT stopped subgroup showed a fall in cell proliferation between core biopsy and operation. The median change in Ki67 (with an interquartile range) for each patient group by OR status is shown. No change was seen in OR-negative tumours across groups (*P*=0.77), whereas OR-positive DCIS from women stopping HRT showed larger changes in proliferation compared to OR-positive tumours in the other groups (*P*=0.02).

).

In the 18 OR-positive DCIS tumours patients who were hormonally manipulated (HRT stopped), a significant reduction in cell proliferation between core biopsy and surgery (mean Ki67 LI on core=9.95%, mean Ki67 % LI at operation=5.06, *P*<0.01) occurred. In OR-positive DCIS from women who continued HRT or were not taking HRT, no change in the mean DCIS cell proliferation between core biopsy and surgery was seen (Table 1).

The hormone manipulated OR-positive DCIS (HRT stopped, *n*=18) were further analysed to assess the response in individual tumours in the group. Despite all DCIS tumours in this subgroup expressing OR (⩾5%), only approximately half the tumours showed a reduction in proliferation during the period of hormone withdrawal, a finding that was independent of nuclear grade (50% high-grade DCIS showed a fall in cell proliferation).

**Figure 1:**
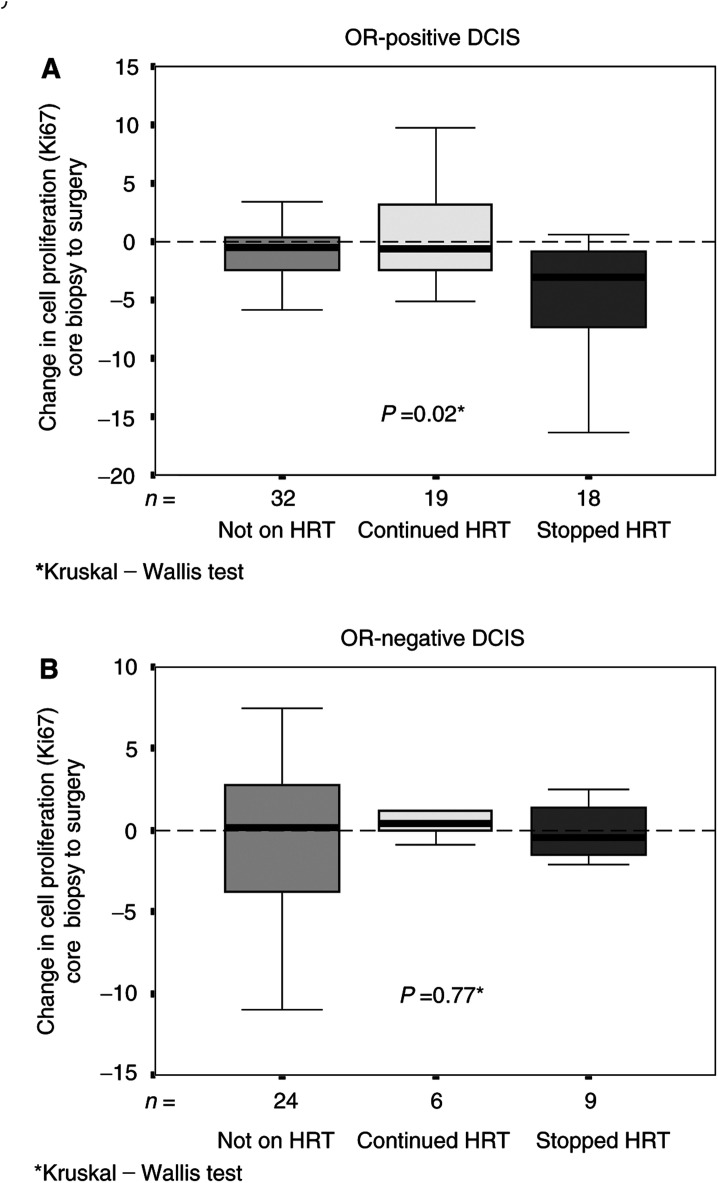
Comparison of the changes in cell proliferation (Ki67) between core biopsy and surgery in the DCIS tumours across groups (stopped HRT, continued HRT and not using HRT) by OR status. For each group, median values are shown as thick horizontal lines, the boxes represent interquartile range and the bars the full range. In OR-positive DCIS tumours (**A**), the median change in proliferation in the stopped HRT is significantly greater than other groups (*P*=0.02, Kruskal–Wallis). There was no difference in the changes in proliferation across groups in OR-negative tumours (**B**), *P*=0.77.

An across-group comparison was made of the magnitude of change in DCIS cell proliferation between core biopsy and surgery according to OR status (Table 1, Figure 1

). In OR-positive DCIS tumours, the fall in proliferation was much greater in group 1 (HRT withdrawal) compared to the other two groups (*P*=0.02). No difference in the magnitude of change in proliferation across the groups was seen in the OR-negative DCIS tumours (*P*=0.73, Table 1, Figure 1).

### Effect of hormone manipulation on PR expression

Progesterone receptor expression was determined on 99 DCIS tumours in this series. Of these, 29 were PR negative and 70 PR positive (stopped using HRT, *n*=19; continued to take HRT, *n*=19; and not using HRT, *n*=32). A significant fall in cell proliferation was observed in women with PR-positive DCIS stopping HRT between core biopsy (mean 11.1, s.e. 1.62) and surgery (mean 6.8, s.e. 1.52, *P*<0.01), but in no other group. However, paired core biopsy/operation expression data were only available for 40 of these because of insufficient core tissue remaining after Ki67 analysis.

Out of the 40 PR-positive tumours with paired data, 14 patients stopped HRT. In these, a significant fall in the mean PR expression was observed between core biopsy (mean, 42.3%) and operation (mean 29.2%, *P*=0.009). No difference in the mean PR expression between core biopsy and surgery was seen in women who continued HRT (*n*=11) or were not taking HRT (*n*=15, Table 2

**Table 2:** Change in PR expression in DCIS between core biopsy and operation

**Patient group (*n*)**	**Mean core PR LI (s.e.)**	**Mean op PR LI (s.e.)**	**95% CI of PR LI difference**	***P*-value^a^**	**Median change in PR LI (IQR)**	***P*-value^b^**
Stopped HRT (14)	46.2 (9.4)	29.2 (7.9)	4.9 to 29.0	0.009	−10.0 (−31.3 to −1.00)	0.002
Continued HRT (11)	55.9 (11.9)	56.5 (12.2)	−4.9 to 3.7	0.75	0.0 (−1.0 to 6.0)	
Not using HRT (15)	42.3 (8.7)	51.5 (8.9)	−19.6 to 1.2	0.08	+5.0 (−4.2 to 16.0)	

a Paired samples *t*-test for change in PR expression between core biopsy and surgery for each subgroup.

b Kruskal–Wallis test for the magnitude of change in PR across groups. LI=Labelling Index (% of nuclei labelled); CI=confidence interval; s.e.=standard error of mean; IQR=interquartile range.

Progesterone receptor-expression data for 40 PR-positive DCIS tumours for which paired sampledata between core biopsy and operation (14–41 days later) were available. Women stopping HRT show a significant fall in DCIS PR expression (*P*=0.009). A mean PR increase in women not using HRT was seen between core biopsy and surgery (*P*=0.08). The magnitude of change in PR expression across the groups was also significant (*P*=0.002).

).

An across-group comparison of the magnitude of change in PR expression between core biopsy and operation groups (Table 2) showed a significant difference between the groups due to the effect of the PR reduction in the HRT-stopped group (*P*=0.002, Kruskal–Wallis). In women who were not using HRT, a nonsignificant increase in PR expression was seen.

Comparing PR and Ki67 change in those patients stopping HRT, the magnitude of reduction in PR expression (median −15.00%) was greater than the magnitude of reduction in Ki67 (median −3.01%, *P*<0.01, Wilcoxon signed-rank test).

### HER-2 expression

This was determined on 92 operative tissue samples. The overall HER-2 positivity was 66%. HER-2 expression was significantly greater in OR- or PR-negative than OR- or PR-positive DCIS (HER-2 positive tumours, 48% OR positive and 58% PR positive; HER-2 negative tumours, 87% OR positive and 93% PR positive (*P*<0.001, Mann–Whitney *U*-test). There was no difference in the median HER-2 expression between tumours in the three patient groups (*P*=0.26, Kruskal–Wallis).

HER-2 expression did not affect response to oestrogen withdrawal (*P*=0.27, Mann–Whitney *U*-test). Specifically, the reduction response in Ki67 cell proliferation between core biopsy and surgery in OR-positive DCIS was seen equally in both HER-2-positive and -negative DCIS (*P*=0.02 and 0.03, respectively, paired samples *t*-test).

## DISCUSSION

The time period between diagnostic core biopsy and operation represents an important window to investigate the effects of drug therapy.

In IBC, OR status predicts response to preoperative therapy in OR-positive cancers treated with tamoxifen (Clarke *et al*, 1993; Chang *et al*, 2000), arimidex (Miller *et al*, 2001; Geisler *et al*, 2001), letrozole (Ellis *et al*, 2001) and faslodex (Robertson *et al*, 2001). In addition, OR status predicts adjuvant response to tamoxifen and is an excellent marker of response to 5 years of therapy, with a reduction in death from IBC of 23% for OR-rich tumours but with no benefit for OR-poor tumours (Early Breast Cancer Trialists' Collaborative Group, 1998).

In OR-positive IBC, changes in cellular proliferation measured by Ki67 nuclear antigen labelling can be used to reliably predict the response to antioestrogen therapy (Clarke *et al*, 1993; Chang *et al*, 2000; Dowsett *et al*, 2001a; Robertson *et al*, 2001; Harper-Wynne *et al*, 2002).

No studies have addressed the biological effect of hormone manipulation on DCIS. In particular, we know of no studies that have examined the effect of a change in HRT on cell proliferation in breast cancer (*in situ* or invasive). However, most clinicians recommend stopping HRT on diagnosis of DCIS, despite the lack of evidence for any biological benefit.

In this study, we have used changes in DCIS cell proliferation and progesterone receptor expression as surrogate markers of likelihood of tumour response to hormonal manipulation to evaluate the potential benefit of stopping HRT in preventing local recurrence after BCS for DCIS based on the OR status of the DCIS tumours.

This was a retrospective, nonrandomised study of consecutive women presenting with DCIS in one unit, and is limited because of small numbers when patients are divided into subgroups. However, we have clearly demonstrated a significant fall in the proliferation in DCIS epithelium following a period of oestrogen withdrawal in OR-positive (but not in OR-negative) tumours, consistent with that reported in OR-positive IBC after treatment with either aromatase inhibitors (Ellis *et al*, 2001; Miller *et al*, 2001) or tamoxifen (Chang *et al*, 2000). Furthermore, PR-expressing DCIS also showed a fall in the level of PR expression (an oestrogen-dependent protein) when HRT was withdrawn. No changes in cell proliferation in DCIS tumours were seen in patients not taking HRT or those continuing HRT during the period between core biopsy and surgery, irrespective of OR status; these patient groups represented internal controls.

The observation that oestrogen withdrawal does not result in a change in cell proliferation in OR-negative DCIS supports our previous findings in a human xenograft mouse model of DCIS demonstrating the hormone independence of OR-negative DCIS (Holland *et al*, 1997). The growth of OR-negative DCIS is driven by activation of the type-1 tyrosine kinase cell surface receptors epidermal growth factor receptor (EGFR) and HER-2 receptor and can be inhibited by EGFR receptor antagonists (Chan *et al*, 2002). This study provides further evidence in patients that OR-negative DCIS is oestrogen independent.

Using the DCIS animal model, we have also shown that apoptosis (programmed cell death, another index of cell turnover) increases in OR-positive, but not OR-negative, DCIS following antioestrogen therapy with the pure antioestrogen, faslodex. However, these apoptotic changes occur earlier than 14 days following treatment, and a recent clinical trial investigating the short-term biological effects of antioestrogen therapy in IBC did not demonstrate any change in apoptosis in the period upto 21 days after treatment with either faslodex or tamoxifen (Robertson *et al*, 2001). Therefore, we did not use apoptosis to assess tumour response to a change in therapy during the 14–41 day period used in this study (Leal *et al*, 1995).

The retrospective analysis of OR status in approximately one-third of DCIS tumours from the NSABP B-24 trial (by case note review and central Immunohistochemical assay) has been confirmed in the findings of this study in the clinical setting, with DCIS OR positivity associated with a significant reduction in the risk of LR after tamoxifen therapy (RR=0.41, confidence interval (CI)=0.25–0.65, *P*=0.0002), while OR-negative tumours showed a nonsignificant benefit in reducing LR from therapy (RR=0.8, CI 0.41–1.56, *P*=0.51). However, the total number of events in the latter group was deemed too small to exclude a small benefit (Allred *et al*, 2002). Since the B-24 trial contained many women with DCIS at the excision margins, these results do not demonstrate the actual benefit of tamoxifen by OR status in reducing LR when DCIS has been completely excised.

The authors of the NSABP B-24 trial (Fisher B *et al*, 1999) recommend tamoxifen (in combination with radiotherapy) for all patients following BCS for DCIS despite the side effects of vasomotor symptoms and potential increased risk of thromboembolic disease and endometrial cancer induced by tamoxifen (Fisher *et al*, 1998). We believe that this recommendation is not biologically (present study) or clinically (pathological review findings of the B-24 trial (Allred *et al*, 2002)) justifiable and needs revision.

HER-2 (CerbB-2/*Neu*) expression in IBC is associated with hormone unresponsiveness, even when OR is expressed (Dowsett *et al*, 2001b). We found no evidence in this study that HER-2 expression in DCIS adversely affects the biological response to oestrogen withdrawal, although HRT (oestrogen) withdrawal is more closely related in nature to aromatase inhibition than to tamoxifen therapy. There have been no trials of adjuvant aromatase inhibition after the treatment for DCIS. This is an area that needs to be addressed, since HER-2 receptor is overexpressed in approximately 60% of DCIS (Leal *et al*, 1995).

Since contralateral breast cancer after treatment for DCIS occurs at a rate of approximately 1% per year for 10 years and since prophylactic tamoxifen therapy in women at high risk for breast cancer reduces DCIS occurrence by 50% and reduces OR-positive (but not OR-negative) IBC occurrence by 69% (Fisher *et al*, 1998), its role as a chemopreventative agent could be used to justify its use in women with OR-negative DCIS to prevent a small number of *de novo* OR-positive cancer occurrences in these high-risk women. However, a recent meta-analysis of the results of adjuvant tamoxifen therapy trials for early IBC found no conclusive data on either survival benefit or reduction in contralateral breast cancer recurrence to support the use of tamoxifen for women with OR-negative breast cancer (Fisher B *et al*, 1998,^1999^).

Only approximately 50% of cases of DCIS are OR positive (Chaudhuri *et al*, 1993). We have shown in this study that only approximately 50% of the OR-positive DCIS respond to oestrogen withdrawal, suggesting that as few as 25% of patients with DCIS may benefit from hormonal manipulation as an adjuvant treatment. The present study was designed to demonstrate the biological response of DCIS to short-term hormone manipulation, not to assess LR risk in this patient cohort; this can only be assessed prospectively in trials using hormone therapy (antioestrogen or hormone replacement) in women where the OR status of the tumours is known.

It is likely that the side-effect profile of long-term tamoxifen use (including pulmonary embolism, deep vein thrombosis and endometrial cancer) will outweigh any clinical benefit for OR-negative DCIS or, indeed, if given to unselected women with DCIS. The UK committee on safety of medicines and the medicines control agency (March 2002) have recommended that tamoxifen should no longer be used for the chemoprevention of breast cancer mainly because of the associated thromboembolic risk (Committee on Safety of Medicines and the Medicines Control Agency, 2002).

Adjuvant hormonal treatment of women with DCIS now needs to be individualised, a goal that has been achieved for IBC and one that we should now aim for in DCIS. For patients with OR-positive DCIS, clinicians should endeavour to enrol as many patients as possible into clinical trials to compare the efficacy of aromatase inhibitors with tamoxifen to profile new relative clinical benefits (e.g. the International Breast Cancer DCIS study II).

This study highlights the biological importance of OR status in predicting response to hormonal therapy for DCIS. At present, the use of HRT after treatment for DCIS should be restricted to women with OR-negative tumours, since these are biologically unresponsive to oestrogen, and therefore HRT therapy should not affect the risk of local recurrence. Oestrogen receptor-negative DCIS has been shown to be hormone independent, thus adjuvant tamoxifen therapy in women with OR-negative DCIS is likely to produce increased morbidity without any clinical benefit. Oestrogen receptor status should now be determined prospectively on all newly diagnosed DCIS and should be used to guide the use of adjuvant hormone therapy.
